# Identifying Factors Associated With Breastfeeding Length Among Filipino Migrant Women in South Korea

**DOI:** 10.1111/jhn.70030

**Published:** 2025-03-10

**Authors:** Winny Dhestina, Heejin Lee, Sherlyn Mae P. Provido, Grace H. Chung, Sangmo Hong, Sung Hoon Yu, Chang Beom Lee, Jung Eun Lee, Zhaoli Dai

**Affiliations:** ^1^ Department of Food and Nutrition, College of Human Ecology Seoul National University Seoul Korea; ^2^ Research, Institute of Human Ecology Seoul National University Seoul Korea; ^3^ Department of Child Development and Family Studies, College of Human Ecology Seoul National University Seoul Korea; ^4^ Division of Endocrinology and Metabolism, Department of Internal Medicine Hanyang University Guri Hospital, Hanyang University College of Medicine Guri Korea; ^5^ School of Population Health, Faculty of Medicine and Health University of New South Wales Sydney Australia; ^6^ School of Pharmacy, Faculty of Medicine and Health University of Sydney Sydney Australia

**Keywords:** breastfeeding, diet, Filipino, Korea, migrants, mothers

## Abstract

**Background:**

Migrant women becoming mothers often face social, economic, and family challenges that can affect their dietary and breastfeeding practices. This study identified factors associated with breastfeeding length in migrant women.

**Methods:**

The study sample involved 504 migrant women from the Filipino Women's Diet and Health Study (FiLWHEL) in 2014–2016. Two‐hundred‐seventy women who had completed information on demographic characteristics, 24‐h dietary recall, breastfeeding, parity, and health conditions were included in the analysis. Multivariable logistic and linear regression models were applied to identify significant factors associated with breastfeeding length cross‐sectionally.

**Results:**

The median (interquartile range [IQR]) for age was 35 (30, 40) years, and the mean body mass index (BMI) was 23.8 kg/m^2^; 62 women (23%) were breastfeeding for at least 1 year, with the median (IQR) length of 4 (1, 10) months per child. The median (IQR) of the total intake of fruits, vegetables, nuts, and legumes was 165.5 (76.9, 265.9) g/day. Women who consumed the highest tertile of fruits, vegetables, nuts, and legumes compared to those in the lowest tertile were more likely to breastfeed for at least 12 months (adjusted‐OR [95% CI]: 2.24 [1.08–4.67]), primarily driven by vegetable consumption (adjusted‐OR [95% CI]: 2.34 [1.11–4.93]). Additionally, women in the highest tertile of these food groups or earned an annual income of 20–40 M KRW (~15–30 K USD) appeared to breastfeed longer compared to their counterparts (*p* < 0.05).

**Conclusions:**

This study suggests that dietary quality and income may impact breastfeeding duration for migrant women in South Korea.

## Summary

- The median breastfeeding length of Filipino migrant women in South Korea was lower than the WHO recommendation, with a median (IQR) of 4 (1, 10) months per child.
- High consumption of fruit, vegetables, nuts, and legumes, particularly vegetable consumption (highest vs. lowest tertile), was associated with a twofold increased likelihood of breastfeeding for 12 months or longer.
- Migrant mothers who earned a mid‐range annual income compared to those who earned less tended to breastfeed longer.

## 1 Introduction

Breastfeeding is vital to infants' health and development. Longer breastfeeding length improves a child's cognitive development [1], educational achievement at 5 years [2], and diet quality at 3 years [3]. Additionally, longer breastfeeding practice has been shown to benefit the mother's health, including lower risks of diabetes and hypertension later in life [4], breast cancer occurrence [5], endometriosis [6], and mortality [7].

Consistent with these health benefits to infants and mothers, the World Health Organization (WHO) and the United Nations Children's Fund (UNICEF) have several breastfeeding recommendations, including early initiation of breastfeeding during the first hour of birth, exclusive breastfeeding during the first 6 months of the infant's life, and continued breastfeeding for up to 2 years of age. However, the prevalence of exclusive breastfeeding by 6 months and breastfeeding continuation for 12 months often fall short of these recommendations in both high‐ [8] and low‐middle‐income countries [9]. For example, data from 51 high‐income countries revealed that 18% of the mothers had exclusively breastfed while 45% had mixed (a combination of breast and formula feeding) or formula‐only feeding by the time their baby turned 6 months. At 12 months, continued breastfeeding decreased to 29% [8]. A review of 57 low‐ to mid‐income countries reports 51.9% for early initiation of breastfeeding, 45.7% for exclusive breastfeeding under 6 months, 32.0% for exclusive breastfeeding at 4–5 months, and 83.1% for continued breastfeeding at 1 year [9].

Additionally, previous studies have suggested that multilevel factors, spanning those in the socio‐cultural context, healthcare system, family and community, workplace and employment, to mother's and infant's attributes, can play a crucial role in sustained breastfeeding practices [10]. For migrant women, new mothers often face higher social, economic, and family challenges that may affect dietary and breastfeeding practices to a larger extent [11]. However, the factors associated with breastfeeding among migrant women remain unclear.

In South Korea, the number of migrants, including Filipino migrant women, has increased nearly threefold, from 4186 in 2006 to 11,673 in 2022 [12]. These women migrated to South Korea through marriage to Korean men and gave birth in the new country. However, research evidence regarding their breastfeeding practices is limited. To address this research gap, a cross‐sectional analysis was conducted to identify factors associated with breastfeeding length in Filipino migrant women in South Korea.

## 2 Methods

### 2.1 The FiLWHEL Cohort

This cross‐sectional study utilized the baseline data from the Filipino Women's Diet and Health Study (FiLWHEL). Filipino migrant women over 19 who had been married to a Korean man were recruited as the study participants. This cohort was designed to understand how diet, lifestyle, environment, and genetics impact their health and the development of chronic diseases in Filipino migrant women in South Korea. Convenience sampling was employed in Filipino communities across various provinces. Data on socioeconomic background, medical history, and health behaviors were captured using a combination of self‐reported and interviewer‐assisted questionnaires. Trained healthcare professionals, overseen by physicians, conducted on‐site anthropometric assessments, blood pressure measurements, and biospecimen collection. The complete design and protocol of the FiLWHEL study have been reported elsewhere [13]. This study adhered to the STROBE reporting guidelines for cross‐sectional studies (Supporting Information).

#### 2.1.1 Study Population

**Figure 1:**
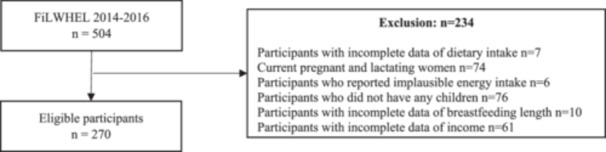
Study participant flow diagram.

The study sample was drawn from the baseline data of the on‐going FiLWHEL cohort. At the study's baseline (2014–2016), 504 participants were recruited and participated in the survey, of whom 270 met the inclusion criteria and were included in the analysis (Figure 1). Those who did not have children (*n* = 76), were pregnant (*n* = 20), were breastfeeding (*n* = 54) at the time of recruitment, and those who did not have complete dietary data (*n* = 7), those who did not report breastfeeding length (*n* = 10) or income information (*n* = 61) were excluded. Participants who reported implausible energy intake above or below ±3 standard deviation of the log‐transformed mean of energy intake (*n* = 6) were also excluded. Log transformation of the energy intake values normalized the distribution of these values.

#### 2.1.2 Outcome: Breastfeeding Length

Based on the reported breastfeeding duration in months and the number of children each woman had, the breastfeeding length for each child was calculated by dividing the total months by the number of children regardless of whether the breastfeeding was exclusive or non‐exclusive. The breastfeeding length variable was then dichotomized into < 12 months and ≥ 12 months for the logistic regression analysis. We also used breastfeeding in months as a continuous variable outcome.

#### 2.1.3 The Main Exposure of Interest: Dietary Assessment

Dietary intake was assessed using the 24‐h recall method through a face‐to‐face interview. Detailed assessments, including time, place, type, amount, and the meal recipe, were conducted by trained researchers. Food photographs, miniatures, and household measures were used as portion‐size demonstrations [13].

Dietary data processing and nutrient value computation were estimated using the computer‐aided analysis program (CAN‐Pro 4.0 for professionals, Korean Society of Nutrition, Seoul, Korea). Foods that were not available in the existing Korean food database were added from the Philippines Food and Nutrition Research Institute (FNRI), the United States Department of Agriculture (USDA), or directly from the nutritional information provided by food manufacturers [14].

According to the nutrient density approach, food intake estimates were presented in grams per 1000 kcal to adjust total dietary caloric intake [15]. The Minimum Dietary Diversity for Women (MDD‐W) score was derived to represent dietary diversity. The MDD‐W score was calculated from ten food groups, with a range of scores from 0 to 10. Consuming more or equal to 15 grams of one food group adds one point. The food groups included in the scoring included (i) grains, white roots, and tubers; (ii) legumes (beans, peas, lentils); (iii) nuts and seeds; (iv) dairy; (v) meat, poultry, and fish; (vi) eggs; (vii) dark green leafy vegetables; (viii) other vitamin A‐rich fruits and vegetables; (ix) other vegetables; and (x) other fruits. The complete information on the MDD‐W scoring has been reported elsewhere [14, 16]. The total consumption of fruit, vegetables, nuts, and legumes was categorized as a combined variable into tertiles, representing fiber‐rich food groups and diet quality [17], and individual dietary variables by tertiles based on each intake value.

#### 2.1.4 Other Covariates: Anthropometrics, Demographics, and Lifestyle Factors

Body weight was measured using the electronic weight scale (InBody 620, Biospace Co. Ltd, Seoul, Korea). Height, waist, and hip circumference were measured using a non‐stretchable measuring tape with a precision of 0.1 cm. A questionnaire in English was administered with the help of Filipino staff to gather information on age in years, marital status, living arrangement, level of education, employment status, insurance status, household income, and Korean language proficiency. For lifestyle factors, cigarette smoking (ever, never), alcohol consumption (drinks per week), sleeping and napping habits (hours or times per week), and moderate physical activity (times per week) were also recorded.

#### 2.1.5 Statistical Analyses

Descriptive statistics of participants' demographic characteristics, anthropometric, and lifestyle factors were presented by the tertiles of total intake of fruits, vegetables, nuts, and legumes and by breastfeeding length (< 12 months; ≥ 12 months).

Univariable and multivariable logistic regressions were used to estimate the associations between participant characteristics and breastfeeding length categories (< 12 months; ≥ 12 months), presented by odds ratios (ORs) and 95% confidence intervals (CIs) for breastfeeding ≥ 12 months. Moreover, univariable and multivariable linear regressions were conducted to assess the relationship of the selected factors with breastfeeding length in months. Variables included in the analyses were age (continuous, years), marital status (married, divorced), living arrangement (alone, with family), education (high school and below, associate/vocational, university and above), employment (employed, others), insurance status (yes, no), income level (< 20 M, 20–40 M, > 40 M Korean won (1 M won is equivalent to US$747)), Korean language proficiency (not well, well), ever smoked (no, yes), current alcohol use (no, yes), moderate physical activity (no, yes), sleep duration (< 5 h, 5–6 h, > 7 h), and fruits, vegetables, nuts and legumes consumption in grams per day (Tertile 1 [lowest], Tertile 2, Tertile 3 [highest]). The median was assumed for continuous variables, and the most frequent category was assigned for categorical variables if participants had missing data ( 5% of the cohort population), including BMI (*n* = 2), waist‐hip ratio (WHR) (*n* = 3), physical activity (*n* = 7), napping duration (*n* = 9), napping frequency (*n* = 10), ever smoking (*n* = 1), and sleeping duration (*n* = 5).

In the bidirectional stepwise regression procedures, variables from the full models that reached a statistical significance of *p* < 0.1 in the multivariable logistic regression models were retained to assess the association with breastfeeding length (< 12 months vs. ≥ 12 months) and the multivariable linear regression models to assess the relationship with breastfeeding length in months.

All statistical analyses were conducted using SAS version 9.4 (SAS Institute Inc., Cary, NC, USA). All reported p‐values were two‐sided; a *p*‐value less than 0.05 was considered statistically significant.

## 3 Results

Among the 270 eligible participants, 62 (23%) breastfed for at least 1 year. Overall, the participants included in the analysis had a median age of 35.5 (interquartile range [IQR]: 30, 40) years, a median breastfeeding length of 4 (IQR: 1, 10) months, and a mean BMI of 23.8 kg/m^2^. The median time between the survey and breastfeeding was 6 (IQR: 3, 11) years. For dietary intake, the median (IQR) of fruits, vegetables, nuts, and legumes was 165.46 (76.98, 265.91) g/day, and the median MDD‐W score of 5 (IQR: 4, 6). Over 50% of the sample (*n* = 157) held a university degree or higher, and 47.8% (*n* = 129) were employed. Most women (90%) were married (*n* = 242) and living with family (*n* = 258). Most women (69%) earned less than 20 million Korean won (~ US$15,000) per year. Over half (*n* = 148) understood Korean as a language well.

The characteristics of participants according to each tertile distribution of the total intake of fruit, vegetables, nuts, and legumes were presented in Table 1. Participants with higher consumption of fruit, vegetables, nuts, and legumes (Tertile 2 and 3) had a longer breastfeeding length of at least 12 months, a higher education level, and more proficiency in Korean than those in Tertile 1. Participants across tertiles had similar proportions of marital status, living arrangement, employment and insurance status, and income level. Similarly, they also had similar BMI, WHR, and moderate physical activity levels. Participants with higher consumption of fruit, vegetables, nuts, and legumes (Tertile 2 and 3) had a higher MDD‐W score than participants in Tertile 1. The higher the consumption of fruit, vegetables, nuts, and legumes, the less likely they smoked tobacco or consumed three drinks or more alcohol per month. Napping duration tended to be higher among participants in the lower tertile of fruit, vegetables, nuts, and legumes consumption.

**Table 1:** Participants' demographic characteristics, anthropometric, and lifestyle factors by tertile distribution of total intake of fruits, vegetables, nuts, and legumes in the FiLWHEL study (*n* = 270).

Characteristics	Tertile 1	Tertile 2	Tertile 3
*N* (number of participants)^c^	90	92	88
Mean ± SD (g/d)	52.41 ± 33.80	168.15 ± 36.06	375.60 ± 170.83
Age (years, mean, SD)	34.40 ± 7.90	35.89 ± 7.29	36.98 ± 7.40
Breastfeeding length^b^ (months, mean, SD)	5.4 ± 6.4	7.6 ± 8.4	7.4 ± 7.8
Breastfeeding categorization (*n*, %)			
< 12 months	76 (84.44)	70 (76.09)	62 (70.45)
≥ 12 months	14 (15.56)	22 (23.91)	26 (29.55)
Marital status (*n*, %)			
Married	82 (91.11)	81 (88.04)	79 (89.77)
Divorced	8 (8.89)	11 (11.96)	9 (10.23)
Living arrangement (*n*, %)			
Alone	3 (3.33)	6 (6.52)	3 (3.41)
Living with family	87 (96.67)	86 (93.48)	85 (96.59)
Level of education (*n*, %)			
High school and below	37 (41.11)	25 (27.17)	22 (25.00)
Associate/vocational	6 (6.67)	9 (9.78)	14 (15.91)
University and above	47 (52.22)	58 (63.04)	52 (59.09)
Employment status (*n*, %)			
Employed	41 (45.56)	45 (48.91)	43 (48.86)
Housewife, self‐employed, unemployed, others	49 (54.44)	47 (51.09)	45 (51.14)
Insurance status (*n*, %)			
Yes	78 (86.67)	84 (91.30)	80 (90.91)
No	12 (13.33)	8 (8.70)	8 (9.09)
Korean language proficiency (*n*, %)			
Not at all or not well	50 (55.56)	35 (38.04)	37 (42.05)
Well or very well	40 (44.44)	57 (61.96)	51 (57.95)
Income level (*n*, %)			
Less than 20,000,000 won	61 (67.78)	63 (68.48)	62 (70.45)
Less than 40,000,000 won	23 (25.56)	23 (25.00)	20 (22.73)
More than 40,000,000 won	6 (6.67)	6 (6.52)	6 (6.82)
BMI^a^ (kg/m^2^, mean, SD)	23.85 ± 3.82	23.88 ± 3.70	23.59 ± 3.83
Waist Hip Ratio (WHR) (mean, SD)	0.82 ± 0.05	0.83 ± 0.06	0.82 ± 0.06
MDD^a^ dietary score (mean, SD)	3.61 ± 1.17	5.28 ± 1.23	5.76 ± 1.33
Moderate physical activity (times/week, mean, SD)	1.40 ± 2.29	1.54 ± 2.26	1.08 ± 2.17
Ever smokers (Yes, *n*, %)	12 (13.33)	7 (7.69)	2 (2.27)
Current alcohol consumption (*n*, %)			
None	30 (33.33)	31 (33.70)	35 (39.77)
Less than or equal to 2 drinks/month	21 (23.33)	18 (19.57)	26 (29.55)
More than 3 drinks/month	39 (43.33)	43 (46.74)	27 (30.68)
Sleeping duration (*n*, %)			
Less than 5 h	17 (19.32)	14 (15.38)	9 (10.47)
Between 5–6 h	36 (40.91)	37 (40.66)	33 (38.37)
More than 7 h	35 (39.77)	40 (43.96)	44 (51.16)
Napping duration (minutes, mean, SD)	28.51 ± 41.53	19.55 ± 36.15	15.83 ± 22.35

^a^ BMI, body mass index; MDD‐W, minimum dietary diversity for women.

^b^ Breastfeeding length was the average length of time in months of breastfeeding per child.

^c^ Some variables had missing data; BMI (*n* = 2), WHR (*n* = 3), physical activity (*n* = 7), napping duration (*n* = 9), napping frequency (*n* = 10), ever smoking (*n* = 1), sleeping duration (*n* = 5).

In Table 2, the participants' demographic and lifestyle factors differed insignificantly according to breastfeeding duration category (< 12 months or ≥ 12 months). In the univariable logistic regression model, a higher MDD‐W score (per 1 score increment) was associated with a higher likelihood of breastfeeding for 12 months or longer. However, it did not reach statistical significance (OR: 1.11 [0.92–1.33]). There were no statistically significant associations for other factors assessed.

**Table 2:** Demographic and lifestyle factors associated with migrant women's breastfeeding for at least 12 months (yes/no, a binary outcome).

Factors	Breastfeeding < 12 months	Breastfeeding ≥ 12 months	Univariable OR^a^ (95% CIs)
Age (years, mean, SD)	35.8 ± 7.6	35.5 ± 7.5	0.99 (0.96–1.03)
Marital status (*n*, %)			
Married	185 (88.94)	57 (91.94)	1.00
Divorced	23 (11.06)	5 (8.06)	0.71 (0.26–1.94)
Level of education (*n*, %)			
High school and below	65 (31.25)	19 (30.65)	1.00
Associate/vocational	21 (10.10)	8 (12.90)	1.30 (0.50–3.41)
University and above	122 (58.65)	35 (56.45)	0.98 (0.52–1.85)
Employment status (*n*, %)			
Employed	98 (47.12)	31 (50.00)	1.00
Housewife, self‐employed, unemployed, and others	110 (52.88)	31 (50.00)	0.89 (0.51–1.57)
Income level (*n*, %)			
Less than 20,000,000 won	146 (70.19)	40 (64.52)	1.00
Less than 40,000,000 won	47 (22.6)	19 (30.65)	1.48 (0.78–2.79)
More than 40,000,000 won	15 (7.21)	3 (4.84)	0.73 (0.2–2.65)
Insurance status (*n*, %)			
Yes	186 (89.42)	56 (90.32)	1.00
No	22 (10.58)	6 (9.68)	0.91 (0.35–2.34)
Korean language proficiency (*n*, %)			
Not at all or not well	98 (47.12)	24 (38.71)	1.00
Well or very well	110 (52.88)	38 (61.29)	1.41 (0.79–2.52)
Ever smoker (*n*, %)			
No	191 (91.83)	57 (93.44)	1.00
Yes	17 (8.17)	4 (6.56)	0.78 (0.25‐2.39)
Alcohol consumption (*n*, %)			
No	70 (33.65)	26 (41.94)	1.00
Yes	138 (66.35)	36 (58.06)	0.90 (0.65–1.24)
Alcohol consumption frequency (*n*, %)			
None	70 (33.65)	26 (41.94)	1.00
≤ 2 times/week	54 (25.96)	11 (17.74)	0.55 (0.25–1.21)
> 3 drinks/month	84 (40.38)	25 (40.32)	0.80 (0.43–1.51)
Sleeping duration (*n*, %)			
< 5 h	32 (15.46)	8 (13.79)	1.00
Between 5–6 h	81 (39.13)	25 (43.10)	1.24 (0.50–3.02)
> 7 h	94 (45.41)	25 (43.10)	1.22 (0.51–2.94)
Napping duration (minutes/week, mean, SD)	20.90 ± 32.15	22.63 ± 42.03	1.00 (0.99–1.01)
Napping frequency (*n*, %)			
None	104 (52.00)	32 (53.33)	1.00
≤ 2 times/week	42 (21.00)	19 (31.67)	1.49 (0.77–2.90)
Between 3–4 times/week	27 (13.50)	4 (6.67)	0.49 (0.16–1.49)
Between 5–7 times/week	27 (13.50)	5 (8.33)	0.61 (0.22–1.71)
MDD‐W^a^ dietary score (mean, SD)	4.83 ± 1.57	5.06 ± 1.46	1.11 (0.92–1.33)
Tertiles of MDD‐W dietary score (*n*, %)			
Tertile 1	87 (41.83)	25 (40.32)	1.00
Tertile 2	46 (22.12)	14 (22.58)	1.06 (0.50–2.23)
Tertile 3	75 (36.06)	23 (37.10)	1.07 (0.56–2.03)

^a^ OR, odds ratio; MDD‐W, minimum dietary diversity for women.

The results of factors associated with breastfeeding duration are described in Table 3. The mean and standard deviation of fruit, vegetables, nuts, and legumes consumption was 188.7 ± 173.5 g/day for participants who breastfed < 12 months and 225.6 ± 141.7 g/day for those who breastfed for at least 12 months. When the intake of fruit, vegetables, nuts, and legumes was investigated as individual variables, the mean of vegetable and legume consumption was higher among those who breastfed for at least 12 months. In the multivariable logistic regression, only physical activity and the combined variable of fruit, vegetables, nuts, and legume consumption were included, as these were the only variables retained based on the pre‐requisite of *p* < 0.1 using the stepwise regression.

**Table 3:** Odds ratio (OR) and 95% confidence interval (CI) for breastfeeding for at least 12 months (yes/no, a binary outcome).

Factors	Mean ± SD (grams/day)	Breastfeeding < 12 months	Breastfeeding ≥ 12 months	Univariable OR (95% CIs)	Multivariable OR (95% CIs)
Combined variable					
Fruits, vegetables, nuts, and legumes consumption^a^					
Mean ± SD (g/day)		188.71 ± 173.53	225.61 ± 141.74	1.00 (1.00–1.00)	
Tertile 1 (*n*, %)	52.41 ± 33.77	76 (36.54)	14 (22.58)	1.00	1.00
Tertile 2 (*n*, %)	168.15 ± 36.06	70 (33.65)	22 (35.48)	1.71 (0.81–3.59)	1.76 (0.83–3.72)
Tertile 3 (*n*, %)	375.60 ± 170.83	62 (29.81)	26 (41.94)	2.28 (1.10–4.73)	2.24 (1.08–4.67)
P for trend				0.03	0.04
Moderate physical activity^a^ (*n*, %)					
Less than once a week		127 (62.87)	45 (73.77)	1.00	1.00
At least once a week		75 (37.13)	16 (26.23)	0.62 (0.33–1.17)	0.62 (0.33–1.19)
Individual variables					
Fruit consumption^b^					
Mean ± SD (g/day)		68.59 ± 140.40	59.60 ± 92.97	0.99 (0.99–1.00)	
Tertile 1 (*n*, %)	0.00 ± 0.00	100 (48.08)	32 (51.61)	1.00	
Tertile 2 (*n*, %)	33.75 ± 22.41	44 (21.15)	11 (17.74)	0.78 (0.36–1.69)	
Tertile 3 (*n*, %)	194.10 ± 177.76	64 (30.77)	19 (30.65)	0.93 (0.49–1.78)	
P for trend				0.88	
Vegetable consumption^a^^,^ b					
Mean ± SD (g/day)		101.00 ± 81.03	136.15 ± 110.56	1.00 (1.00–1.01)	
Tertile 1 (*n*, %)	24.71 ± 18.75	72 (34.62)	13 (20.97)	1.00	1.00
Tertile 2 (*n*, %)	89.37 ± 20.90	72 (34.62)	22 (35.48)	1.69 (0.79–3.62)	1.78 (0.83–3.83)
Tertile 3 (*n*, %)	208.22 ± 78.71	64 (30.77)	27 (43.55)	2.34 (1.11–4.91)	2.34 (1.11–4.93)
P for trend				0.03	0.03
Nut consumption^b^					
Mean ± SD (g/day)		0.91 ± 4.41	1.77 ± 6.05	1.03 (0.98–1.09)	
Nonconsumer (*n*, %)	0.00 ± 0.00	166 (79.81)	48 (77.42)	1.00	
Consumer (*n*, %)	5.35 ± 9.55	42 (20.19)	14 (22.58)	1.15 (0.58–2.23)	
P for trend				0.68	
Legume consumption^b^					
Mean ± SD (g/day)		8.20 ± 29.63	28.08 ± 43.60	1.01 (1.00–1.02)	
Tertile 1 (*n*, %)	0.00 ± 0.00	96 (46.15)	24 (38.71)	1.00	
Tertile 2 (*n*, %)	5.31 ± 3.70	38 (18.27)	11 (17.74)	1.16 (0.52–2.59)	
Tertile 3 (*n*, %)	52.15 ± 37.24	74 (35.58)	27 (43.55)	1.46 (0.78–2.74)	
P for trend				0.25	
Moderate physical activity^a^ (*n*, %)					
Less than once a week		127 (62.87)	45 (73.77)	1.00	1.00
At least once a week		75 (37.13)	16 (26.23)	0.62 (0.33–1.17	0.61 (0.32–1.16)

^a^ The variable with *p* < 0.1 in the stepwise approach was included in the multivariable models: moderate physical activity (less than once a week/at least once a week), a combined variable of fruits, vegetables, nuts, and legumes consumption (Tertile 1–3), and individual variable of vegetable (Tertile 1–3).

^b^ The individual variables of fruits, vegetables, nuts, and legume consumption were entered simultaneously and included in the multivariable model. However, only moderate physical activity (less than once a week/at least once a week) and vegetable consumption were retained in the multivariable model.

Participants at the highest tertile intake compared with the lowest tertile intake had an over twofold likelihood to breastfeed for 12 months or longer (adjusted‐OR [95% CI]: 2.24 [1.08–4.67]). Only two variables, physical activity and vegetable consumption, were retained. Higher vegetable consumption (highest tertile vs. lower tertile) was associated with breastfeeding for at least 12 months (adjusted‐OR [95% CI]: 2.34 [1.11–4.93]), while higher legume consumption showed a suggestive but nonsignificant association (adjusted‐OR [95% CI]: 1.46 [0.78–2.74]). Weekly moderate physical activity was not associated with the outcome accessed.

In Table 4, the results from the linear regression analysis were presented. The variables included in the models were income level (< 20 M, 20‐40 M, > 40 M KRW), the combined variable of fruit, vegetables, nuts, and legumes consumption in tertiles, and vegetable consumption in tertiles. For every unit increase, those whose consumption of fruit, vegetables, nuts, and legumes in the second tertile were more likely to breastfeed longer than those who consumed the lowest tertile of food intake (beta‐coefficient: 2.23; SE: 1.12; *p* = 0.05). Similarly, women who consumed more vegetables (third tertile) were more likely to breastfeed longer than the lower tertiles (beta‐coefficient: 2.16; SE: 1.14; *p* = 0.06). Additionally, women who earned an annual income between 20 and 40 million Korean won were more likely to breastfeed longer than the reference group who earned less than 20 million won after adjustment for fruit, vegetables, nuts, and legume intake as a combined variable (beta‐coefficient: 2.85; SE: 1.08; *p* = 0.01) or vegetable intake in the regression models (beta‐coefficient: 2.98; SE: 1.09; *p* = 0.01).

**Table 4:** Linear regression using beta coefficients and standard errors for length of breastfeeding (months, continuous variable).

Factors	Beta coefficient	Standard error	*p*‐value
Combined variable			
Fruits, vegetables, nuts, and legumes consumption^b^ (Mean ± SD, g/day)			
Tertile 1 (52.41 ± 33.77)	Ref		
Tertile 2 (168.15 ± 36.06)	2.23	1.12	0.05
Tertile 3 (375.60 ± 170.83)	2.11	1.13	0.06
Income level^b^			
Less than 20,000,000 won	Ref		
Between 20,000,000 and 40,000,000 won	2.86	1.08	0.01
More than 40,000,000 won	−0.45	1.86	0.81
Individual variables			
Fruit consumption^b^ (Mean ± SD, g/day)			
Tertile 1 (0.00 ± 0.00)	Ref		
Tertile 2 (33.75 ± 22.41)	−0.51	1.23	0.68
Tertile 3 (194.10 ± 177.76)	0.80	1.07	0.46
Vegetable consumption^a^^,^ b (Mean ± SD, g/day)			
Tertile 1 (24.71 ± 18.75)	Ref		
Tertile 2 (89.37 ± 20.90)	0.69	1.13	0.54
Tertile 3 (208.22 ± 78.71)	2.16	1.14	0.06
Nuts consumption^b^ (Mean ± SD, g/day)			
Nonconsumer (0.00 ± 0.00)	Ref		
Consumer (5.35 ± 9.55)	0.01	1.15	0.99
Legumes consumption^b^ (Mean ± SD, g/day)			
Tertile 1 (0.00 ± 0.00)	Ref		
Tertile 2 (5.31 ± 3.70)	−0.92	1.30	0.45
Tertile 3 (52.15 ± 37.24)	0.66	1.03	0.52
Income level^a^			
Less than 20,000,000 won	Ref		
Between 20,000,000 and 40,000,000 won	2.98	1.09	0.01
More than 40,000,000 won	−0.40	1.86	0.83

^a^ The variable with *p* < 0.1 in the stepwise approach was included in the multivariable models: moderate physical activity (less than once a week/at least once a week), a combined variable of fruits, vegetables, nuts, and legumes consumption (Tertile 1–3), and individual variable of vegetable (Tertile 1–3).

^b^ The individual variables of fruits, vegetables, nuts, and legume consumption were entered simultaneously included in the multivariable model, but only moderate physical activity (less than once a week/at least once a week) and vegetable consumption were retained in the multivariable model.

## 4 Discussion

This study was the first to explore breastfeeding‐associated factors among Filipino migrant women in South Korea. In this cross‐sectional analysis, women with a higher dietary intake of total fruits, vegetables, nuts, and legumes were more likely to breastfeed their child for at least 1 year, indicating the vital role of diet quality in breastfeeding length in these migrant women. The observed association between the combined intake of fruit, vegetables, nuts, and legumes and breastfeeding length in this population appears to be driven primarily by vegetable consumption. Legume consumption may also play a role, but further investigation is needed to confirm this. Most participants in our study held a university degree or higher, and nearly half were employed. Most women were married and lived with family, while the majority earned less than 20 million Korean won per year. These findings suggest that education and employment may contribute to healthier dietary choices due to increased nutrition knowledge, resource access, and financial stability. However, further research is needed to establish causal relationships and explore the long‐term impact of these factors on dietary behaviors and breastfeeding outcomes.

In this study, we adopted the approach from the WHO and Food and Agriculture Organization (FAO) guidelines [16, 17] by using dietary intake of fruit, vegetables, nuts, and legumes to represent diet quality. Our results suggest that a higher intake of fruit and vegetables was significantly associated with longer breastfeeding length. Similar to this finding, mothers who had a higher adherence to the Mediterranean diet, characterized by higher dietary intakes of fruits, vegetables, nuts, and legumes, produced more nutritious breastmilk, including higher contents of antioxidants and proteins than mothers with a lower Mediterranean diet adherence [18]. In other studies, mothers with higher consumption of vegetables were found to have breastmilk with higher contents of vitamin A and carotenoids [19]. Additionally, mothers who consumed more fruit produced a higher abundance of human milk oligosaccharides (HMOs), known to have promotive effects on neonatal digestive, immune, and nervous system development [20]. This line of evidence implies that a mother's diet quality, especially with increased fruit and vegetable intake, may improve the quality and quantity of breastmilk, leading to longer breastfeeding length and, consequently, better health outcomes in their offspring [21].

This analysis also suggests that migrant women's annual income between 20 and 40 million Korean won (approximately US$15,000–30,000) was positively associated with a longer breastfeeding length than those with lower income (less than 20 million Korean won or US$15,000). However, no association was found among those who earned over 40 million Korean won. This finding indicates that jobs in the middle‐income bracket may provide sufficient financial support and possibly lower work pressure, which may contribute to better physical and mental well‐being, enhanced milk production, stronger mother–infant bonding, better sleep quality, and increased maternal confidence [22], potentially leading to more breastfeeding time for these migrant mothers. Previous studies have suggested that a higher household income of ≥ US$30,000 in Canada and > US$1405 in China tended to lengthen breastfeeding in working mothers, while those who earned < US$30,000 in Canada and < US$702 in China did not breastfeed or had a shortened length [23, 24]. This evidence on the mother's income suggests that sufficient income is necessary to support maternal nutrition and diet quality due to the relatively higher price of fresh fruit and vegetables. In the previous study conducted among US women, mothers who did not consume adequate vegetables earned a lower income than those who had an adequate intake [25].

Aside from the dietary and financial factors associated with a more extended breastfeeding period, other social factors, such as family support, have also been suggested as crucial. For example, a systematic review of qualitative studies among Asian, Latino, and African migrants in high‐income countries (USA, Europe, and Australia) suggested that family and friends' influence was essential to their breastfeeding practices [11]. In the same vein, a study among migrant Chuukese mothers in Guam reports that lack of support from family and community was a significant barrier to breastfeeding [26]. Moreover, support from spouses, elderly relatives, and friends was associated with longer breastfeeding length in Chinese mothers [24]. In this study, most participants lived with their family (95.6%), including 70.7% with their spouses and 16.3% with their parents or parents‐in‐law. While a certain level of family support for breastfeeding was assumed, this study suggests that individual factors such as diet quality and income level are significant factors associated with breastfeeding duration.

Finally, a shorter breastfeeding length was recognized among Filipino mothers who migrated to South Korea than those who stayed in their own country. The median breastfeeding length in this study was 4 months, similar to a median of 5 months in the 2018 National Survey on Fertility and Family Health and Welfare results in South Korea [27]. By contrast, the mean length of breastfeeding was 8.3 months among Filipino mothers in their own country, according to the Philippine National Nutrition Survey in 2019 [28]. However, breastfeeding length is generally longer in low‐income and middle‐income countries than in high‐income countries [29]. Given the substantial health benefits of breastfeeding to mothers and babies, awareness and support must be advocated widely among all mothers, including migrant women. For example, South Korea has implemented lactation facilities in public places and workplaces to encourage breastfeeding outside of homes, providing residents access to lactation education, counseling services, and support groups to residents [30, 31].

There were several strengths of the study. First, this study had a unique population of Filipino migrant women who migrated to South Korea based on marriage. Understanding their health and nutrition provides a breadth of knowledge in dietary patterns and socioeconomic spectrums. Second, the data were collected using a standardized protocol, including double‐checking by trained data enumerators to minimize measurement errors in the data. Future research should understand the health and development of the offspring and the mental and physical well‐being of these mothers related to the breastfeeding practice in these migrant women.

This study also included several limitations. First, the findings may not infer a causal relationship, drawing findings from a cross‐sectional study. Second, the results may not represent the general population as a convenience rather than a random sampling method was adopted. Third, a single 24‐h dietary recall to obtain the dietary data may not fully capture the participants' usual dietary habits. Fourth, the small sample size can limit the study's statistical power, potentially leading to false negative results. These limitations highlight the need for caution in interpreting the findings. Finally, self‐reported data on diet and breastfeeding length were prone to misclassification. However, the ability of mothers to recall pregnancy‐related events that occurred 30 or more years ago, including breastfeeding, was found to be reproducible and valid in participants of the Nurses' Health Study [32]. Given the current study's limitations, further investigations in larger cohorts using longitudinal designs, random sampling, and multiple dietary assessments are warranted to provide a more robust understanding of the findings. Finally, the generalizability of the findings may be limited to migrant women in South Korea.

## 5 Conclusion

This study highlights the pivotal role of diet quality and financial independence in fostering a sustained breastfeeding experience in Filipino women who migrated to South Korea. These findings underscore the need for a comprehensive, multi‐faceted approach to supporting and promoting breastfeeding practices for migrant women, such as food assistance programmes, health education, nutrition literacy, as well as family and workplace support systems.

## Author Contributions

W.D., J.E.L., and Z.D. conceptualized the study. J.E.L. and C.B.L. obtained funding for the research. H.L., S.M.P.P., G.H.C., S.H., S.H.Y., C.B.L., and J.E.L. collected the data. W.D., H.L., and S.M.P.P. conducted the analysis. W.D. and Z.D. drafted the manuscript. All authors reviewed and approved the final version to be published and agreed to be accountable for the work.

## Ethics Statement

The study was approved by the Institutional Review Board (IRB) of Sookmyung Women's University (SMWU‐1311BR‐012). All participants provided written informed consent upon participating in the study; their personal information remained deidentified in the secondary data analysis.

## Conflicts of Interest

The authors declare no conflicts of interest.

### 1 Peer Review

The peer review history for this article is available at https://www.webofscience.com/api/gateway/wos/peer-review/10.1111/jhn.70030.

## Supporting information

Supporting information.

## Data Availability

The data used in this study cannot be made publicly available because FiLWHEL is an on‐going study, and participants were not informed during the consent process that their information would be stored in a publicly accessible database. Requests to access the data may be sent to the data access committee (nutepid@gmail. com), Dr. Jung Eun Lee (jungelee@snu. ac. kr), and Dr. Chang Beom Lee (lekang@hanyang. ac. kr).
